# Facilitators and barriers primary care physicians face in providing palliative care in low- and middle-income countries: a mixed methods systematic review

**DOI:** 10.1186/s12875-025-03023-5

**Published:** 2025-12-29

**Authors:** GVC Fernando, L Athauda, TK Perdamaian, JS Kondasinghe, S Prathapan

**Affiliations:** 1 University of Sri Jayewardenepura, Nugegoda, Sri Lanka; 2 University of Kelaniya, Kelaniya, Sri Lanka; 3 Universitas Kristen Duta Wacana, Yogyakarta, Indonesia; 4 University of Western Australia, Perth, Australia

**Keywords:** Primary care physicians, General practitioners, Palliative care, Developing countries, Primary care, Primary health care

## Abstract

**Background:**

Primary care physicians play a crucial role in delivering palliative care, particularly in low-and middle-income countries, where access to specialist services is often limited. However, various facilitators and barriers influence their ability to provide effective palliative care.

**Objective:**

This systematic review aimed to identify and synthesise the facilitators and barriers experienced by primary care physicians in low- and middle-income countries when providing palliative care.

**Methods:**

A convergent integrated mixed-methods review adhered to PRISMA guidelines. Seven databases (MEDLINE, CINAHL, PsycINFO, Scopus, Google Scholar, Policy Commons, and ProQuest) were searched. Data were synthesised using reflexive thematic analysis and narrative synthesis, and quantitative findings were summarised descriptively.

**Results:**

Twelve studies met the inclusion criteria. Five overarching themes were identified: (1) health system organisation, (2) coordinating and sharing care responsibilities, (3) primary care physicians’ disposition, (4) effects on physicians and their regulation, and (5) interacting with patients and families. Key barriers included poor infrastructure, shortages in staff, medicines and funding, lack of clear referral systems, insufficient training, emotional burden, legal uncertainties and cultural or religious resistance to palliative care. Facilitators included supportive health reforms, interdisciplinary teamwork, integration with specialist services, home-based care models, motivated clinicians and opportunities for practical, ongoing education.

**Conclusion:**

The findings highlight the urgent need for targeted interventions, including enhanced training, policy reforms, and improved resource allocation, to strengthen the role of primary care physicians in palliative care in low- and middle-income countries. Future research should focus on context-specific solutions to address these barriers and improve palliative care accessibility in resource-limited settings.

**Supplementary Information:**

The online version contains supplementary material available at 10.1186/s12875-025-03023-5.

## Background

Palliative care aims to enhance the quality of life of individuals facing life-threatening illnesses and their families, and it constitutes a crucial component of healthcare systems worldwide [1]. The World Health Organization (WHO) advocates integrating palliative care within primary healthcare (PHC) systems to improve accessibility, particularly in low- and middle-income countries (LMICs). Primary care, often the first point of contact in healthcare, is increasingly recognised as a key setting for ensuring universal access to essential palliative care [2–4].

Primary care physicians (PCPs) in LMICs face unique challenges in delivering palliative care, including limited resources, inadequate training and systemic barriers [5, 6]. Despite these challenges, successful models of integration exist, often driven by community engagement and locally adapted care frameworks (Atreya et al., 2019; Pai et al., 2023). However, disparities persist, with palliative care services in high-income countries benefiting from better integration, robust policies and structured training programs [7]. In contrast, LMICs struggle with inadequate healthcare infrastructure, limited access to essential medications and societal stigma surrounding end-of-life care, further hindering PCPs’ ability to provide comprehensive palliative care [8–10]. Inadequate PCP numbers also hamper palliative care delivery in primary care settings. Recent data show that in LMIC regions such as Sub-Saharan Africa and South Asia, physician density is often ≤ 10 per 10,000 population, while nurse and midwife density typically ranges from 9 to 20 per 10,000, with nurse-to-doctor ratios around 8:1 [11, 12]. This substantial numerical predominance of nursing cadres reinforces arguments that, within a task-shifting agenda toward palliative care in primary care settings, investing in nurse-centred training may yield greater reach and impact than focusing on physicians.

A key challenge in LMICs is the lack of formal palliative care training and clear guidelines for PCPs [13, 14]. Many report feeling underprepared to address complex palliative needs, including symptom management, end-of-life communication, and psychosocial support. Consequently, palliative care delivery in primary care remains inconsistent, leaving many patients without adequate end-of-life care [15].

This systematic review explores the facilitators and barriers PCPs encounter when providing palliative care in LMICs. Key themes shaping palliative care provision in these settings are identified by synthesising empirical literature. Understanding these factors is vital for integrating palliative care into existing healthcare frameworks and improving policy and practice. Additionally, this review provides insights into systemic, sociocultural, and personal factors influencing PCPs’ ability to deliver quality palliative care. The findings contribute to ongoing discussions on improving palliative care access in LMICs and serve as a foundation for future research, including an empirical study examining the experiences of Sri Lankan general practitioners.

### Review design

The systematic review protocol was registered with PROSPERO (CRD42024573046). A convergent integrated mixed-methods review approach facilitated the integration of empirical evidence from diverse methodologies, offering a comprehensive understanding of facilitators and barriers while identifying commonalities and discrepancies [16]. Given the subjectivity and contextual variability of physicians’ perceptions of facilitators and barriers, an interpretivist epistemological lens was adopted [17]. Reflexive thematic analysis and narrative synthesis were applied to maintain interpretive flexibility while balancing inductive and deductive reasoning [18]. This approach aimed to inform practice, policy and evidence gaps [19].

Alternative mixed methods review approaches were considered but deemed unsuitable. The integrative review approach [20] was too broad, while framework synthesis lacked relevant established frameworks [21]. Critical interpretive synthesis was not adopted as it focuses on theory refinement rather than exploring existing facilitators and barriers [22].

## Methods

The review adhered to PRISMA guidelines [23], encompassing question formulation, search execution, eligibility criteria definition, literature retrieval, critical appraisal, data extraction, analysis, synthesis, discussion and reporting. The review question was framed on the PICOS framework (Methley et al., 2014), as detailed in Table 1.

**Table 1:** Components of the review question and their description

Attribute	*Study design*
*Population*	Physicians providing first-contact primary care.
*Intervention*	Delivery of palliative care.
*Context*	Primary care settings in low- and middle-income countries
*Outcomes*	Facilitators and barriers physicians perceive in providing palliative care within primary care settings.
*Study design*	Empirical studies of qualitative, quantitative or mixed methods design, including grey literature.

### Search strategy

A systematic search was conducted across seven databases from 1 July 1999 through 31 December 2024 inclusive: MEDLINE, CINAHL, PsycINFO (via EBSCOhost), Scopus, Google Scholar, Policy Commons, and ProQuest Dissertations and Theses. Appendix 1 outlines the search strategies conducted in the respective databases. The strategy incorporated database-specific subject headings, wildcards and curated text words. Screening of search results followed a stepwise process, prioritising relevance. Supplementary searches included expert consultations, citation tracking and reference list screening of nine related systematic reviews (Appendix 2). Searches concluded on 31 December 2024.

###  Eligibility criteria and literature retrieval

Eligibility criteria are outlined in Table 2. Studies were imported into Covidence [24] for deduplication and systematic screening. The primary reviewer (CF) screened titles and abstracts, followed by full-text reviews, with a random 25% of full texts independently reviewed by secondary (LA) and tertiary (TP) reviewers. Disagreements were resolved through consensus.

**Table 2:** Eligibility criteria for study inclusion

Criterion	Inclusion criteria	Exclusion criteria
Study design	• Empirical studies (qualitative, quantitative or mixed-methods research designs) • Research revealing PCPs' first-hand experience.	• Participant observations • Commentaries by third parties.
Publication Type	• Peer-reviewed journal publications • Research theses and grey literature.	• Position statements • Guidelines • Policy papers
Population	• Physicians providing first-contact primary care regardless of whether they possess a postgraduate qualification or the level and discipline of such qualification.	• Studies involving physicians whose primary discipline is palliative care • Studies not concerning primary care physicians.
Intervention	• Primary palliative care functions: - identifying people with palliative care needs, - holistically assessing them and - planning and coordinating care • Interventions that cover patients', families’ or caregivers’ physical, psychological, social, and spiritual dimensions	• Studies not revealing the functions of palliative care in primary care settings.
Context	• Primary care settings of countries currently classified by the World Bank as having low-income, low-middle-income, and upper-middle-income economies • Studies regarding physicians rendering their service through individual or group clinics in outpatient, community or home settings.	• Studies regarding secondary or tertiary care settings, whether in the private or public sector • Studies focusing on high-income countries.
Outcome	• Facilitators and barriers directly perceived and expressed by physicians in providing palliative care within primary care settings. • Factors influencing perceptions of physicians situated in different socioeconomic and cultural contexts.	• Facilitators and barriers that cannot be disaggregated by primary care physicians.
Language	• English	• Any other languages
Time window	• Publications within the past 25 years were included. (i.e., 1 July 1999 to 31 December 2024)	--

### Critical appraisal of literature

The Mixed Methods Appraisal Tool (MMAT_Ver_18) was used for quality assessment [25]. Initial screening ensured relevance based on research questions and data suitability. Studies were then assessed for five study design-specific criteria, thus evaluating their methodological rigour, including bias and overall credibility. Independent appraisals by CF and LA were reconciled through discussion. In addition, MMAT_2011 quality ratings were applied [26]. In this step, upon a study fulfilling 5/5 criteria, a rating of 100% was given.

### Extraction

The data extraction form was an adaptation of that developed by the Joanna Briggs Institute [27]. The developed form (Appendix 3) included the study title, author, year, title, aim(s), methods, setting, primary care physician description, other respondents in the study and the aspect of palliative care discussed. Only findings directly attributable to primary care physicians or unanimous expressions across all participants were extracted. Descriptive and statistical results were extracted from the phenomena of interest, themes, categories, quotes from qualitative studies and information from mixed-methods studies. The contextual data of each study were extracted in breadth and depth to enable interpretation of converging or diverging findings across settings. The form was piloted with five studies and refined accordingly.

### Data analysis and synthesis

NVivo (Version 15) facilitated analysis [28]. A convergent integrated mixed-methods review approach was used [29]. Data extracted from studies of disparate designs were analysed in parallel in a single synthesis. Codes were developed iteratively, capturing meaning units. Ongoing refinements were made as new insights emerged, ensuring that the coding framework remained responsive to the data. Given the predominantly qualitative lens of the research inquiry, the quantitative data were ‘qualitised’ into narrative descriptions. For instance, the quantitative finding in the paper by Hertanti and colleagues (2020, page 4), ‘the stepwise multiple regression analysis revealed that 82.9% of provider comfort with caring for terminally ill patients was explained by their experience in caring for terminally ill patients in PHC centres’ [30] was qualitised into ‘better provider comfort through ample experience in caring for terminally ill patients within primary health care’.

Coding of the original qualitative data and qualitised quantitative data followed a reflexive thematic analysis approach, with themes tabulated for comparison. Reciprocal and refutational translations identified similarities and contradictions, forming an analytical matrix. Given the heterogeneity of the quantitative study methodologies, a meta-analysis was not feasible; instead, relevant descriptive statistics (averages, percentages, correlations and frequencies) were presented separately in a table. Sensitivity analyses were used to assess risk of bias from missing or selectively reported results.

## Findings

### Study screening and selection

**Fig. 1:**
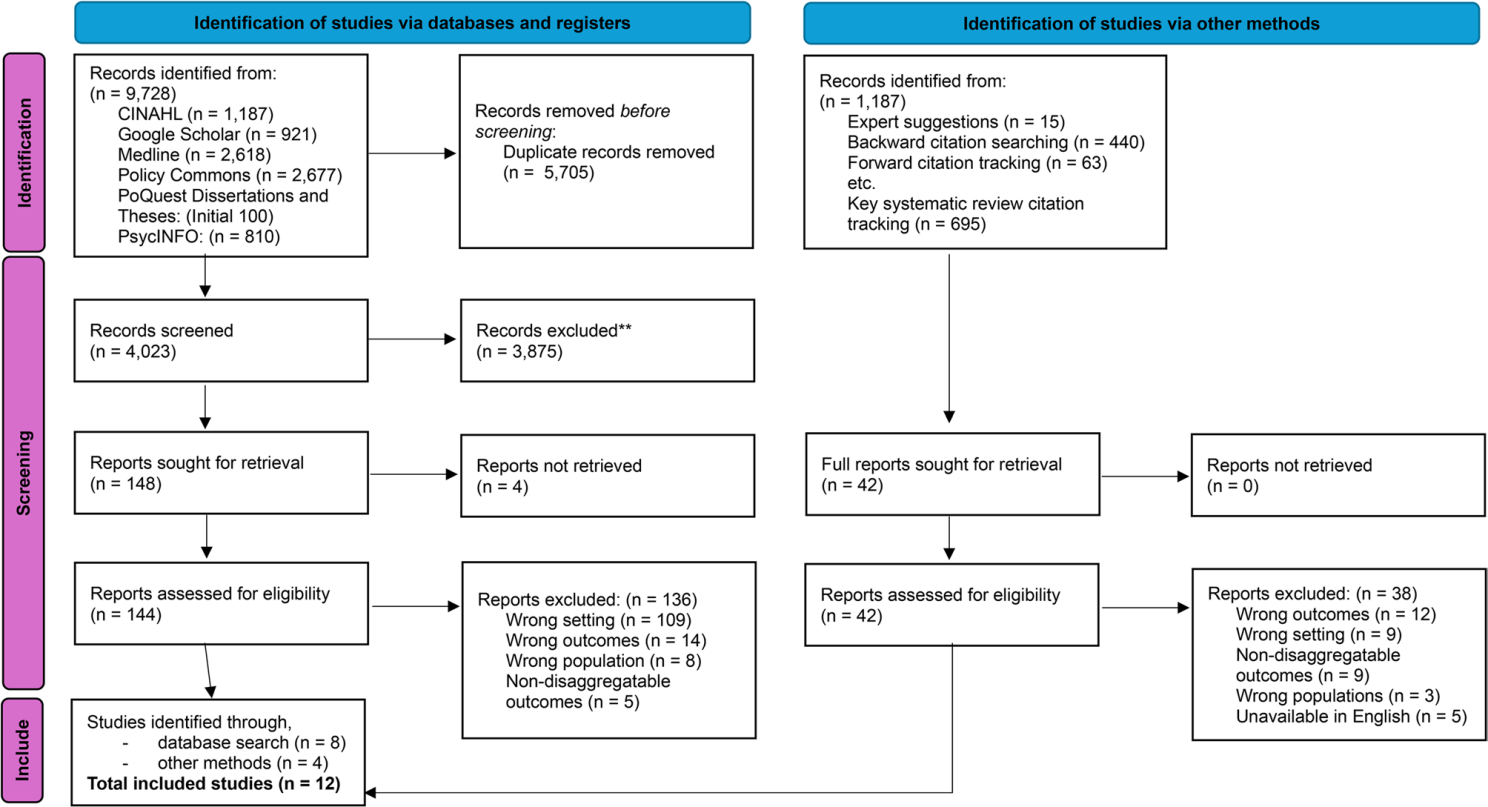
PRISMA 2020 flow diagram for new systematic reviews, which included searches of databases, registers and other sources. Source: Page et al., (2021)

Findings were synthesised within the existing evidence base, with implications for clinical practice, policy, and future research identified. A comprehensive search across seven databases yielded 9,728 records. Covidence removed 5,705 duplicates, leaving 4,023 for screening. After title and abstract screening, 3,875 records were excluded, and 148 full texts were assessed, with eight meeting the inclusion criteria. An additional 1,187 records were identified through other sources, of which four were eligible. Ultimately, twelve studies were included. The selection process is summarised in Fig. 1.

### Overview of included studies

The review comprises twelve studies involving 722 primary care physicians across rural, semi-urban, and urban settings in seven LMICs: Albania (*n* = 1), China (*n* = 1), India (*n* = 3), Indonesia (*n* = 1), Iran (*n* = 4), Nigeria (*n* = 1), and Thailand (*n* = 1). Eight studies used qualitative methods, while four employed descriptive quantitative approaches. Five studies focused on general palliative care provision, four on home and community-based care, and three each on pain management, rural healthcare, and critical incidents. Table 3 provides detailed study characteristics.

**Table 3:** Overview of the study characteristics

Author and year	Title	Aim(s)	Methods	Setting	Primary care physician description.	Other respondents in the study.	Aspect(s) of palliative care discussed
Afolabi et al., 2022 [31]	Integrated primary palliative care in Nigeria- perspectives of patients, families and providers	To identify preferences and expectations for primary palliative care among people with serious illness and their families and the readiness of primary healthcare providers to deliver primary palliative care in Nigeria.	Cross-sectional qualitative descriptive semi-structured interview study, underpinned by the pragmatist approach to identify and solve problems. Data were analysed using thematic analysis.	Three primary healthcare centres, one urban, one rural-urban and one rural, in Ibadan, Nigeria.	Three primary care physicians with at least six months experience in primary health care.	Patients and family caregivers attending specialist outpatient clinics (i.e., palliative care, cardiology, neurology, pulmonology, and cancer radiotherapy outpatient units) at a national referral teaching hospital.	General palliative care provision.
Asadi-Lari et al., 2009 [32]	The concept of palliative care practice among Iranian general practitioners	To assess general practitioners’ knowledge about palliative care to develop special palliative care educational programmes	Cross-sectional quantitative survey using a questionnaire.	Primary care settings in rural and urban areas in Iran.	Two hundred and sixteen general practitioners attending a continuous medical education programme.	Not applicable.	General palliative care provision.
Atreya et al., 2019 [6]	Integrated primary palliative care model; facilitators and challenges of primary care/family physicians providing community‑based palliative care	To understand the facilitators and challenges in liaison networking and delivering palliative care as perceived by primary care/family physicians.	Exploratory quantitative survey.	Home care service of the Tata Medical Centre in Kolkata, India.	One hundred family physicians and primary care physicians offering home care services.	Not applicable.	Home care
Atreya & Mondal, 2024 [33]	Healthcare professionals’ perspectives on factors influencing integration of primary palliative care into mainstream health service: an exploratory focus group study	To explore factors that facilitate or hinder palliative care provision.	Focused group interviews analysed using constructivist grounded theory.	District and sub-divisional hospitals in West Bengal, India.	Two doctors are trained in providing palliative care.	One deputy chief medical officer of health, two nurses, and one epidemiologist.	General palliative care provision.
Damani et al., 2018 [34]	Exploring education and training needs in palliative care among family physicians in Mumbai: A qualitative study.	To explore the extent of knowledge of family physicians about palliative care and the need for additional training.	Qualitative exploratory analysis of content leading to theme and theory development.	Home care services rendered by tertiary cancer centres by employing family physicians in four suburbs of Mumbai, India.	Ten family physicians (average age, 51, and 30% women), all remaining up to date with continuous medical education.	Forty per cent of the participants were Ayurvedic physicians.	Home care services.
Dehi et al., 2021 [35]	Barriers to end-of-life care delivery to home-dwelling terminally-ill older patients: A qualitative content analysis.	To explore the barriers to end-of-life care delivery to home-dwelling terminally ill older patients.	In-depth, semi-structured, face-to-face interviews analysed through conventional content (qualitative) analysis.	Homes, home care institutes and hospitals that provide home care for terminally ill, old patients in Tabriz, Iran	Four physicians who had experience in end-of-life care delivery to home-dwelling, old, terminally ill patients.	Five nurses, one nurse assistant and ten family caregivers.	Home care.
Hertanti et al., 2020 [20]	Knowledge and comfort related to palliative care among Indonesian primary health care providers.	To assess the knowledge and comfort of Indonesian primary health care providers in relation to palliative care	Descriptive cross-sectional quantitative design using Palliative Care Quiz for Nursing (PCQN) – Indonesia tool to assess knowledge and Visual Analogue Scale to assess comfort levels.	Primary health care centres in Yogyakarta, Indonesia.	One hundred forty-three physicians (71% female) work in primary care centres. Sixty-four per cent have received formal palliative care education.	Three hundred and seventy-three nurses, of whom less than a quarter had experience in palliative care (n ¼ 121; 23.4%).	General palliative care provision.
Hojjat-Assari et al., 2022 [36]	Explaining health care providers’ perceptions about the integration of palliative care with primary health care; a qualitative study.	To explain health care providers’ perception of integrating palliative care with primary health care.	Twenty-one semi‑structured interviews and one focused group session followed by a conventional qualitative content analysis approach.	Comprehensive urban and rural health centres providing primary health care in Iran.	One general practitioner and one non-specialist medical doctor.	Oncologists, palliative care specialists, nurses, psychologists, social workers, and primary health care system experts.	Home and community-based palliative care.
Jabbari et al., 2018 [37]	Organising palliative care in the rural areas of Iran: are family physician-based approaches suitable?	To explore the experiences and views of family physicians and other key stakeholders about the feasibility of organising palliative and end-of-life care in rural areas of Iran.	Two focus group discussions, which were analysed through a (qualitative) thematic analysis approach.	Peripheral units of the health system providing preventive health and other primary health care services in rural areas of Iran	Twenty-three rural family physicians with at least three years of work experience and seen at least three patients who needed palliative care in the previous two years.	Thirteen other key palliative and end-of-life care stakeholders.	Rural palliative care provision.
Lai et al., 2018 [38]	The experience of caring for patients at the end-of-life stage in non-palliative care settings: a qualitative study	To explore the experiences of health care providers in caring for patients at the end-of-life stage in non-palliative care settings.	Semi-structured individual interviews were analysed through qualitative content analysis.	Community health centres in Shanghai, China.	Two general practitioners who had cared for adult patients at the end-of-life stage during the past two years.	Findings primarily concern physicians and nurses of other acute and subacute non-palliative care settings.	General palliative care provision.
Lertrattananon et al., 2019 [39]	Does medical training in Thailand prepare doctors for work in community hospitals? An analysis of critical incidents	To explore doctors’ perceptions of their preparedness for working effectively in community hospitals in Thailand.	Qualitative thematic analysis of reports of two critical incidents that had occurred while working in a community hospital.	Community hospitals in different regions of Thailand.	Twenty-eight family medicine residents. Seventeen had previously trained through a rural track programme, and 11 through a standard track.	Not applicable.	Critical incidents in palliative care.
Xhixha et al., 2013 [40]	Reducing the barriers to pain management in Albania: results from an educational seminar with family doctors.	This study evaluated the attitudes of family doctors on pain assessment, management, and opioid usage before and after seminars on opioid pain management.	The Barriers Questionnaire II (BQ-II) (quantitative) was used to evaluate attitudes towards pain management.	Family doctors-run clinics in rural and urban areas of six cities in Albania.	One hundred and eighty-nine family doctors (general practitioners) who are representatives of the National Association for Palliative Care and from Regional Health Directories.	Not applicable.	Pain management in primary care clinics.

### Critical appraisal of included studies

All twelve studies met the two core MMAT_18 criteria: having clear research questions and data alignment with research objectives. Seven qualitative studies satisfied all five quality criteria, except for Dehi et al.‘s (2021), which showed potential interpretive bias. The four quantitative studies had limitations in sample representativeness and non-response bias due to convenience sampling. Table 4 summarizes the critical appraisal results.

**Table 4:** Critical appraisal findings

Study		Qualitative studies					
		Is the qualitative approach appropriate to answer the research question?	Are the qualitative data collection methods adequate to address the research question?	Are the findings adequately derived from the data?	Is the interpretation of results sufficiently substantiated by data?	Is there coherence between qualitative data sources, collection, analysis and interpretation?	Quality rating
1	Afolabi et al. (2022) [31]	Yes	Yes	Yes	Yes	Yes	100%
2	Atreya & Mondal, (2024) [33]	Yes	Yes	Yes	Yes	Yes	100%
3	Damani et al. (2018) [34]	Yes	Yes	Yes	Yes	Yes	100%
4	Dehi et al. (2021) [35]	Yes	Yes	Yes	No	No	60%
5	Hojjat-Assari et al. (2022) [36]	Yes	Yes	Yes	Yes	Yes	100%
6	Jabbari et al. (2018) [37]	Yes	Yes	Yes	Yes	Yes	100%
7	Lai et al., 2018) [38]	Yes	Yes	Yes	Yes	Yes	100%
8	Lertrattananon et al. (2019) [39]	Yes	Yes	Yes	Yes	Yes	100%
		Quantitative descriptive studies					
		Is the sampling strategy relevant to address the research question?	Is the sample representative of the target population?	Are the measurements appropriate?	Is the risk of non-response bias low?	Is the statistical analysis appropriate to answer the research question?	
9	Asadi-Lari et al. (2009) [32]	Yes	No	Yes	No	Yes	60%
10	Atreya et al. (2019) [6]	Yes	No	Yes	No	Yes	60%
11	Hertanti et al. (2020) [20]	Yes	No	Yes	Can’t tell	Yes	60%
12	Xhixha et al. (2013) [40]	Yes	No	Yes	Can’t tell	Yes	60%

### Facilitators and barriers to palliative care provision within primary care as perceived by primary care physicians:

**Fig. 2:**
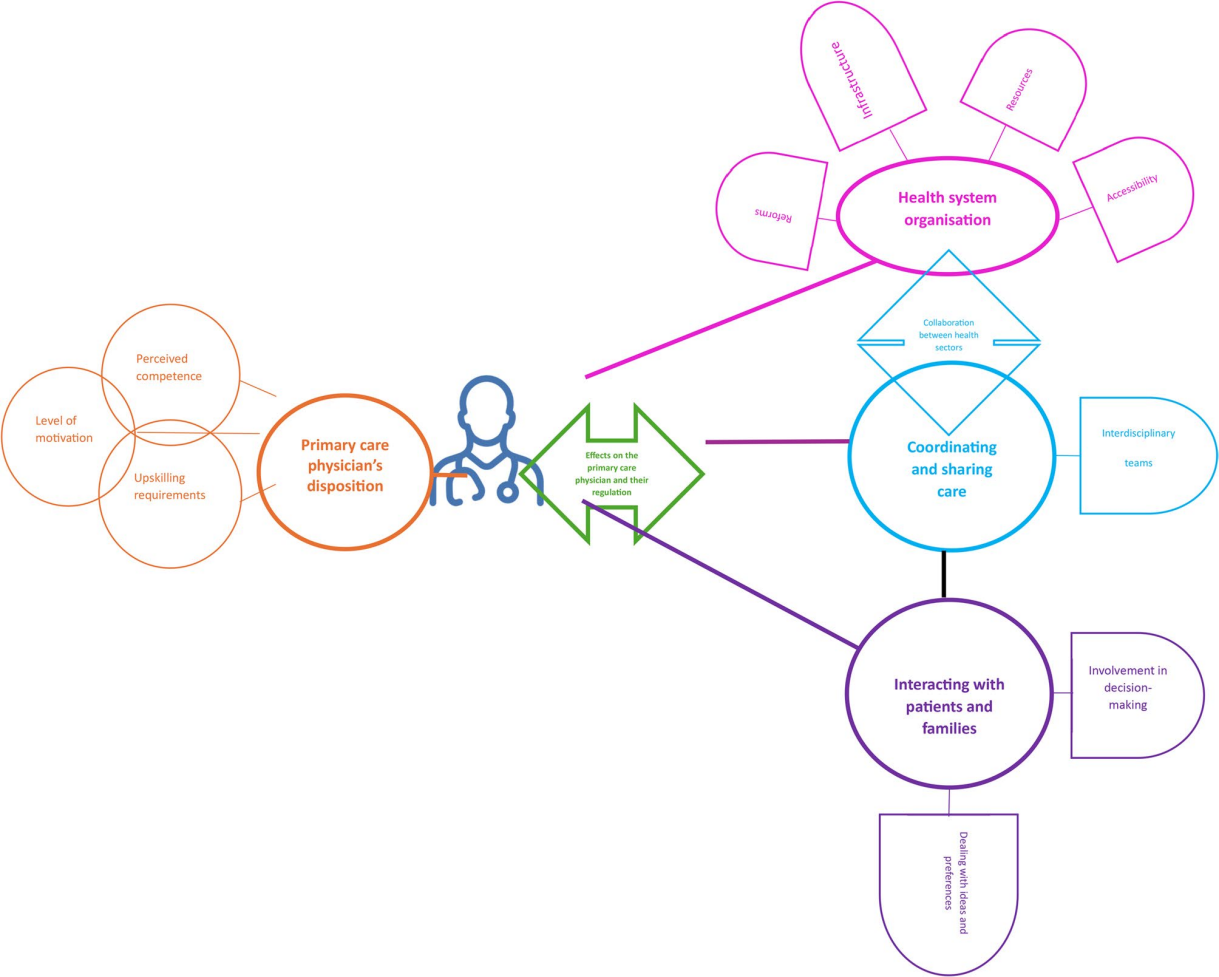
Thematic diagram illustrating relevant themes, subthemes and their relationships

Five overarching themes were iteratively developed: health system organisation, coordinating and sharing care responsibilities, primary care physicians’ disposition, effects on the primary care physicians and their regulation and interacting with patients and families. Four themes were further subdivided into subordinate themes, presented in detail below. The following thematic descriptions capture the breadth and depth of individual themes and subthemes while also being attentive to the convergences and divergences within themes. The thematic map Figure 2 below illustrates the themes, subthemes and likely relationships between them.

1. Health system organisation: Several aspects of the overall health system in which the primary care physician is located influence the ability to deliver palliative care. Four subthemes were iteratively identified: healthcare infrastructure, resource availability, accessibility to palliative care and health system reforms.
  - Healthcare infrastructure: Different papers from Iran revealed that existing capacious primary healthcare system infrastructure serves as an efficient medium for palliative care delivery [36, 37], while poor infrastructure bars optimum care [35]. Atreya & Mondal (2024) identified the degree of administrative support and effort acknowledgement to correspond with the degree of facilitation perceived by physicians. Home-based care was generally regarded positively as a key aspect of quality care [6, 34, 36]. However, in Damani et al.‘s study (2018), the perceived threats and time constraints were pointed out as drawbacks of homecare. Two Indian studies identified liaison with well-equipped hospitals, community healthcare providers and volunteers to strengthen the home care services provided by primary care physicians [33, 34]. Where primary care lacked the focus on long-term care and out-of-hours services, it served as a poor medium for palliative care delivery [6, 31]. Hojjat-Assari et al. (2022) revealed that electronic health records integrated into the mainstream health system are instrumental in comprehensive care provision.
  - Resource availability: Three key forms of resources determined the ability to implement palliative care services. Consistency of fiscal resource allocations is pivotal for the sustainability of healthcare delivery, and problems arise when interruptions in funding occur [33, 34, 37]. Shortages in human resources, poor inter-professional staffing ratios, and the resultant time constraints are pointed out as hindering quality care [31, 33–35]. In terms of physical resources, limited availability of facilities and medications to manage specific life-limiting illnesses hampers the provision of palliative care in the primary care setting [31, 33, 35–38]. One specific limitation revealed in Atreya & Mondal’s (2024) study is the lack of a dedicated space in busy public sector clinics to discuss sensitive matters with patients and families.
  - Accessibility of palliative care: Despite the availability of palliative care, its accessibility to patients is crucial. In contrast to hospitals, regional primary care settings promote easy, timely and less costly access to palliative care for patients within their vicinity with minimal disruptions to their worsening health status [34, 36]. Similarly, the patients’ access to palliative care is also hampered by its unaffordability [6, 33, 35–37]. The erratic supply of medicines through the public sector leads to patients incurring an out-of-pocket expense to source them from private suppliers, thus impeding symptom management. Primary care physicians perceive that for palliative care and medicines to be accessible, they must be free of charge, subsidised or covered by insurance.
  - Health system reforms: Favourable healthcare reforms enhance palliative care delivery within primary care [33, 34, 36, 37]. One reform constitutes integrating palliative care within the national health policy frameworks and public health services as an essential component. Another is decentralising palliative care delivery, ideally by involving primary care professionals. Moreover, the identification of gaps and improvement of services through clinical audits and the participation of healthcare professionals in policy decisions are particularly beneficial [33, 34]. However, stagnant policies that constrain financing or limit full-time healthcare services restrain palliative care delivery [35].
2. Coordinating and sharing care responsibilities: How the care for patients and families is coordinated and shared between different health sectors and personnel determines the palliative care delivery within primary care. Two subthemes were iterated: interdisciplinary teams and care coordination between health sectors.
  - Interdisciplinary teams: Several attributes were desired in relation to the structure and function of interdisciplinary teams. Leadership, a framework for team operation, team members with diverse expertise, cohesiveness, amicable relationships among members and mutual respect where everybody’s voice is heard in decision-making are beneficial for efficient, holistic care [31, 33, 36, 37]. Lack of clear delineation of roles, staff incompetencies and irresponsible behaviours contribute to poor care [31, 37]. On the contrary, poor training and competencies among team members in palliative care and poor communication led to counterproductive tensions within such teams [31, 34, 35, 37].
  - Coordination of care between health sectors: Poor coordination between primary health care with other levels of the public health system, home care services, private sector and non-government services act as a barrier to palliative care provision [6, 31, 33, 34, 36]. A formal referral and back referral system is felt to address this problem. Underestimation of the primary care providers’ skills by other professionals, not receiving referrals for non-malignant life-threatening illnesses, and fragmentation of care between health sectors lead to poor care [31, 33]. When special palliative care packages are offered in collaboration between different health sectors, also integrating psychosocial services, better care can be anticipated [34, 37].

Seamless liaison with specialist palliative care professionals or services empowers the palliative care provision within primary care [33, 34, 36, 37]. Written agreements catered for individual patients’ care between specialists and primary care providers are specifically beneficial [6, 36]. As the bidirectional referral systems between palliative care specialists and primary care physicians fail, care provision becomes suboptimal.

3. Primary care physicians’ disposition: Primary care physicians’ inherent disposition determines their practice and potential to deliver palliative care within primary care. Three subthemes identified were their perceived competence, level of motivation and upskilling requirements.
  - Perceived competence: The physicians’ perceived competence in palliative care in terms of knowledge, skills and experience were instrumental in palliative care provision [6, 30, 31,32, 34, 37, 39]. Specific competencies including but not limited to assessment and management of symptoms, communication skills and dealing with children and their parents enhanced the physicians’ confidence in implementing palliative care in their primary care practice [6, 32, 33, 39].
  - Level of motivation: The degree to which the primary care physician is motivated to provide palliative care, among other care responsibilities, translates into actual care provision. While some consider palliative care as part of their duties, others express no interest in integrating it into their routine care [35, 37]. The physicians’ motivations were to develop their palliative care competencies and help the patients to their best abilities [6, 31, 34]. Atreya & Mondal’s (2024) study proposes that physicians’ self-actualisation as their patients improve with treatments and when patients appreciate them motivates them further.
  - Upskilling requirements: The specific areas the physicians wished to develop were essential palliative care competencies, communication and compassion [31, 33, 34, 39, 40]. Interprofessional training programmes, seminars, continuous professional development events, case-based discussions and hands-on sessions are preferred to distance learning opportunities [33, 34, 40]. Lack of clinical and ethical guidance serves as an obstacle to patient management, and physicians wish to be familiar with clear guidelines as part of their clinical competence to deliver appropriate care [33–35].
4. Effects on the primary care physician and their regulation: The provision of palliative care has many implications for physicians. Caring for patients with life-threatening illnesses, especially children, which invariably entails dealing with death and dying, can be a psychologically overwhelming process that results in burnout among physicians [30, 31, 33–35]. These studies also reveal that an increased workload may lead to work-life imbalance. Damani et al. (2018) suggest that the lack of palliative care training may deteriorate physicians’ well-being. However, Hertanti et al.‘s (2020) study identified that although the duration of exposure to palliative care may improve comfort in dealing with such patients, training may not necessarily do so. Furthermore, the fear of the associated legal implications hinders primary care physicians from opting for palliative care provision [31, 36].

Physicians lean on or wish to employ several measures to mitigate these deleterious effects on them and sustain their duties. Psychological support from peers and, on occasions, from religious leaders and other organisations assist the physicians to minimise such setbacks [33, 34]. However, these physicians expect more formal pathways to be in place for psychosocial support. In addition, physicians aim to self-regulate their well-being and functioning amidst adversities through self-care routines such as time off work, although excessive workloads prohibit such activities [33, 34].

5. Interacting with patients and families: Physicians’ interactions with patients and families govern their ability to implement palliative care. Two subthemes iterated were dealing with patients’ and families’ ideas and preferences and involving patients and families in decision-making.
  - Dealing with patients’ and families’ ideas and preferences: The overall limited public awareness about the practice and principles of palliative care results in the patients and families not readily accepting this approach to care [6, 33, 35]. Similarly, Xhixha et al. (2013) reveal that some individuals are opiophobic, attributing to their adverse effects and addiction potential, thus adding to the problem. The same study also points out a reluctance among patients to discuss symptoms with physicians for many reasons, including a fear that this may disrupt the focus on curative treatment. These studies show that improving public awareness may alleviate these misconceptions and increase acceptance of palliative care.

Having to cater for the preferences of patients and families also affected the physicians’ ability to provide necessary care [33, 35–37]. Some patients averse to death and prefer treatment of curative intent over palliation. Some others prefer to endure pain and suffering based on the religious or cultural belief that symptoms are meaningful towards the end of life. Patients’ preference for in-person consultations over virtual ones and cultural preference to see same-gender physicians are some other barriers, especially where staff are limited. However, physicians feel that providing compassionate, value-for-money services catering to the patient’s needs may enhance their acceptance of palliative care [33, 36].

b. Involving patients and families in decision-making: Physicians generally feel empowered to provide palliative care when they understand the patients, families and their needs, thus establishing therapeutic relationships [33, 36]. Some physicians feel at ease in engaging in conversations, educating patients and families and giving them some control over the decision-making process [6, 33, 37]. When their demands are unreasonable, physicians tend to decide alone denying patient or family involvement [33, 35]. In addition, Dehi et al.‘s (2021) study reveals that tensions and financial constraints within families invariably negatively influence aspects of patient care and decision-making.

### Findings of the quantitative studies

Given the heterogeneity of quantitative studies regarding their specific designs and contexts, their findings did not aggregate towards a meta-analysis. Nevertheless, the relevant quantitative findings were qualitised and integrated into the thematic iterations presented above. Further, Table 5 below summarises the pertinent quantitative figures with context.

**Table 5:** Summary table of relevant quantitative findings

Quantitative study	Pertinent findings
Asadi-Lari et al. (2009) [32]	Twelve per cent of the respondents rated their palliative care knowledge as excellent or very good. At the same time, more than 50% scored as weak or less, significantly related to their previous experience caring for a terminally ill patient (*p* = 0.001). Less than a third stated their ability to assess pain, and a little over a third could manage pain at the end of life.
Atreya et al. (2019) [6]	The proportion of physicians expressing specific barriers and facilitators; Barriers: a. lack of knowledge in palliative care (32%) and b. confidence (34%), c. non‑affordability of home visit service for patients (67%), d. poor out‑of‑hours support (89%) e. poor communication from hospital services (87%) f. unclear information on goals of care from the specialists (87%), and g. poor knowledge about local palliative care services (15%) Facilitators: a. willingness to support palliative care patients (46%) b. discussing advanced care plan with patient/family (21%) c. accessing web resources for knowledge (32%) d. willingness to be trained in primary palliative care (75%) and e. providing home visits (49%)
Hertanti et al. (2020) [30]	Comfort level with caring for terminally ill patients was negatively correlated with the age of health care providers (r ¼~0.191, P, 0.001) and years of work experience in primary health care (r ¼~0.134, P, 0.01). Primary health care providers without experience in caring for terminally ill patients had lower comfort levels than those who had such experience (t ¼~31.7, P, 0.001). The findings also showed that palliative care training did not affect their comfort levels (t ¼~0.29, P. 0.05). However, this variable was included in the stepwise regression analysis because three previous studies’ findings supported its correlation. Accordingly, the regression analysis revealed that 82.9% of the providers’ comfort with caring for the patient at end-of-life could be explained by their previous similar experience, palliative care training and the duration of experience in primary health care.
Xhixha et al. (2013) [40]	The barriers to optimal cancer pain management were assessed using the Barriers Questionnaire II (BQ-II). It is a standardised questionnaire containing 27 items loading on four factors: physiology (12 items), fatalism (3 items), communication (6 items), and harmful effects (6 items). All of the questions were scored on a numerical scale ranging from 0 (do not agree at all) to 5 (agree very much). Major barriers, among others, to accessing palliative care were regarded as those with mean scores of > = three on the scale. They were the preconceptions that ‘many people with cancer get addicted to pain medicine’ (4.0), ‘there is a danger of becoming addicted to pain medicine’ (4.0), ‘using pain medicine blocks your ability to know if you have any new pain’ (3.9), ‘when you use pain medicine your body becomes used to its effects and pretty soon it won’t work anymore’ (3.7), ‘if you take pain medicine when you have some pain, then it might not work as well if the pain becomes worse’ (3.7), ‘pain medicine is very addictive (3.5), ‘it is easier to put up with pain than with the side effects that come from pain medicine (3.1), ‘pain medicine can keep you from knowing what’s going on in your body’ (3.0).

## Discussion

This systematic review provides a comprehensive synthesis of the facilitators and barriers primary care physicians encounter in LMICs when delivering palliative care. The thematic analysis of qualitative studies and the synthesis of quantitative findings offer a nuanced understanding of these settings’ multifaceted challenges and enablers across five overarching themes: health system organisation, coordinating and sharing care responsibilities, primary care physicians’ disposition, effects on the primary care physicians and their regulation, and interacting with patients and families.

### Comparison with existing literature

The results align with and extend key literature, including the Lancet Commission report by Knaul and colleagues (2018) that discussed aspects of palliative care access in LMICs and previous systematic reviews on primary palliative care [41]. The current systematic review’s findings are closely analogous to those of previous reviews, which were predominantly based on evidence arising from high-income settings involving primary care practitioners in general, not specific to physicians. Thematic development in the present review closely resembles that of Rhee and colleagues’ (2020) review: patient factors, personal factors of the PCP, general practice factors, relational factors, coordination of care and availability of services [42]. Similarly, the review by Carey and colleagues (2019) revealed common barriers in bureaucracy, communication among healthcare staff, and primary care professionals’ personal commitments and competencies [43]. The enablers identified were education, trained respite staff to aid care delivery, good interprofessional communication and templates to facilitate referral to out-of-hours services.

Munday and colleagues’ (2024) systematic review synthesised evidence from previous reviews to map comprehensively the personal, system, policy and organisational challenges and possible facilitators for palliative care services specifically for cancer patients in LMICs [5]. Although the aspects identified did not directly reflect the physicians’ perspectives, they shared several elements with the current review: knowledgeability and attitudes among clinicians and the public, adequacy of trained staff, resource availability and national policies. Further, in their integrative review, Silva and colleagues (2021) revealed a pivotal role in managing and organising healthcare services and educational interventions in the discipline [44]. This review, too, did not base its findings on PCPs’ first-hand experiences.

The findings of the present review corroborate those of Mitchell and colleagues (2018), who indicated that inadequate training in palliative care is a significant barrier for primary care physicians [45]. A lack of formal education and continued professional development opportunities limits physicians’ ability to provide high-quality palliative care, as reported in previous reviews focusing on LMICs [41]. Studies from high-income countries have also identified the need for more structured training, but the issue is particularly severe in resource-limited settings where palliative care is not well-integrated into primary healthcare curricula [46]. Interdisciplinary palliative care training programmes, such as the African study reported by Yennurajalingam and colleagues (2025), have shown promise in LMICs [47].

Fernando and Perdamaian (2024), in their editorial, suggested a possible scarcity of essential palliative care resources, including medications, infrastructure and supportive care services, that hamper palliative care delivery in low- and middle-income settings in the Asian context [48]. The suggested deficits have been demonstrated in the current systematic review. Furthermore, our findings align with those of Rajagopal and colleagues (2017), who emphasised that opioid availability remains a critical issue in LMICs, contributing to suboptimal symptom management [49]. Despite WHO guidelines advocating for improved opioid accessibility [50], practical implementation remains insufficient due to regulatory restrictions and sociocultural apprehensions.

Cultural influences also played a significant role in shaping palliative care delivery. As reported in previous research, physicians in LMICs often face challenges related to patients’ and families’ reluctance to discuss death and end-of-life care [51]. Our review reinforces the importance of cultural sensitivity and the need for context-specific interventions that respect local beliefs while promoting effective and pragmatic palliative care discussions. Further exploration is warranted on how the strategies for communication of sensitive information emerging from high-income settings [52] could be appropriated to LMICs.

A theme unique to the current review is the effects of palliative care provision on the PCP and their regulation. These aspects were not discussed in the reviews leaning on high-income countries, arguably as palliative care services form a core function of primary care in many developed settings [44], the practitioners receive the appropriate training as part of their clinical training [53] and availability of psychological support for clinicians through the mainstream health systems [54, 55]. In contrast, in many LMICs where palliative care provision remains sporadic and it may be optional for clinicians to deliver palliative care, they may feel poorly prepared and supported to render their services.

In addition to the patient factors identified in Rhee and colleagues’ (2019) review [42], our findings show profound family involvement in decision-making and caring. This finding may reflect families’ prominent roles in palliative care delivery models in LMICs, as revealed in the systematic review by Peeler and colleagues (2024) [56]. Further, the systematic review by Senior and colleagues (2024) that examined the primary care clinicians’ self-reported multidisciplinary end-of-life care in a global context revealed factors that hindered or enabled advance care planning [57]. This aspect was not evident in the current study, possibly due to limited avenues for advance care planning in LMICs to date. Similarly, our review did not capture the element of out-of-hours access to palliative care, as evident in Carey and colleagues’ (2019) review [43], which might still constitute a developing area in LMICs.

### Implications for policy and practice

The findings underscore the urgent need for health policy reforms to support primary care physicians in providing palliative care. Governments and healthcare institutions should prioritise integrating palliative care into primary healthcare systems through national policies, workforce training initiatives and improved funding mechanisms. Experts propose that when palliative care is embedded into primary healthcare systems, patient outcomes improve, and healthcare costs decrease [58]. Therefore, policymakers should adopt a holistic approach that addresses both systemic and individual-level barriers. Moreover, interdisciplinary collaboration and task-sharing strategies have been suggested as potential solutions to workforce shortages in palliative care [59]. Given the larger workforce of nurses and other health professionals in comparison to physicians in LMICs [12], supporting primary care to deliver palliative care effectively and meaningfully may entail empowering its nurses and community health workers rather than just physicians. Further, it is required to review drug regulatory frameworks and legislation to enable suitably trained nursing and other staff to, for example, prescribe opioid medication.

### Strengths and limitations

This systematic review offers a robust synthesis of evidence by incorporating findings from both qualitative and quantitative studies, allowing for a comprehensive understanding of the topic. The thematic analysis approach provided an in-depth exploration of the complex interactions between individual, institutional system-level and societal factors affecting palliative care delivery. However, several limitations should be acknowledged. First, the variability in study methodologies and limited representation of settings may limit the generalizability of findings across all LMICs. Additionally, the reliance on English-language publications may have excluded relevant studies published in other languages. It is also acknowledged that nurses largely lead primary palliative care in LMICs in Africa [56, 60], which this systematic review has not captured. Lastly, the cross-sectional nature of most included quantitative studies limits the ability to determine causal relationships between identified barriers and facilitators.

### Future research directions

Further research should focus on evaluating the effectiveness of context-specific interventions designed to improve primary palliative care in LMICs. Implementation science approaches could be valuable in assessing the feasibility and scalability of training programs, policy reforms and resource allocation strategies. Additionally, longitudinal studies examining the long-term impact of interventions on patient and provider outcomes would enhance our understanding of sustainable solutions.

## Conclusion

This review highlights the critical barriers and facilitators influencing primary care physicians’ ability to provide palliative care in LMICs. While significant challenges remain, targeted interventions in training, resource allocation, and policy development can substantially improve palliative care delivery in these settings. Integrating palliative care into primary healthcare systems and fostering interdisciplinary collaboration are essential steps toward enhancing access and quality of care for patients with life-limiting illnesses in resource-limited contexts.

## Appendix 1: search strategies for all databases

**Table:** MEDLINE

Population and context	Intervention	Context ⇓	Outcome
primary care physician	palliative care		facilitators and barriers
MH “primary care physician” OR TI (((“primary care” OR “primary health care” OR medic* OR family OR community) N3 (physician OR doctor OR practitioner OR staff OR provider)) OR “general practitioner” OR GP OR PCP OR “family medicine specialist”) OR AB (((“primary care” OR “primary health care” OR medic* OR family OR community) N3 (physician OR doctor OR practitioner OR staff OR provider)) OR “general practitioner” OR GP OR PCP OR “family medicine specialist”)	MH (“palliative care” OR “terminal care” OR “hospice care” OR “palliative medicine”) OR TI (((palliative OR terminal OR “end of life” OR hospice OR eol) N3 (management OR care OR medicine OR treatment))) OR AB (((palliative OR terminal OR “end of life” OR hospice OR eol) N3 (management OR care OR medicine OR treatment)))		TI (facilitat* OR enabler* OR motivat* OR assist* OR help* OR influenc* OR barrier OR bar* OR hinder OR hindrance* OR challeng* OR obstacle OR difficult* OR issue* problem OR hurdle OR imped* OR limit* OR deter* OR constrain* OR obstruct*) OR AB (facilitat* OR enabler* OR motivat* OR assist* OR help* OR influenc* OR barrier OR bar* OR hinder OR hindrance* OR challeng* OR obstacle OR difficult* OR issue* problem OR hurdle OR imped* OR limit* OR deter* OR constrain* OR obstruct*)

Filtered by: English language, Human, 1 July 1999 – 31 December 2024

**Table:** CINAHL

Population and context	Intervention	Context ⇓	Outcome
primary care physician	palliative care		facilitators and barriers
MH (“primary health care” OR “family physicians”) OR TI (((“primary care” OR “primary health care” OR medic* OR family OR community) W3 (physician OR doctor OR practitioner OR staff OR provider)) OR “general practitioner” OR GP OR PCP OR “family medicine specialist”) OR AB (((“primary care” OR “primary health care” OR medic* OR family OR community) W3 (physician OR doctor OR practitioner OR staff OR provider)) OR “general practitioner” OR GP OR PCP OR “family medicine specialist”)	MH (“palliative care” OR “palliative medicine”) OR TI (((palliative OR terminal OR “end of life” OR hospice OR eol) W3 (management OR care OR medicine OR treatment))) OR AB (((palliative OR terminal OR “end of life” OR hospice OR eol) W3 (management OR care OR medicine OR treatment)))		TI (facilitat* OR enabler* OR motivat* OR assist* OR help* OR influenc* OR barrier OR bar* OR hinder OR hindrance* OR challeng* OR obstacle OR difficult* OR issue* problem OR hurdle OR imped* OR limit* OR deter* OR constrain* OR obstruct*) OR AB (facilitat* OR enabler* OR motivat* OR assist* OR help* OR influenc* OR barrier OR bar* OR hinder OR hindrance* OR challeng* OR obstacle OR difficult* OR issue* problem OR hurdle OR imped* OR limit* OR deter* OR constrain* OR obstruct*)

Filtered by: English language, Human, July 1999 – December 2024

**Table:** PyschINFO

Population and context	Intervention	Context ⇓	Outcome
primary care physician	palliative care		facilitators and barriers
MA “primary health care” OR TI (((“primary care” OR “primary health care” OR medic* OR family OR community) W3 (physician OR doctor OR practitioner OR staff OR provider)) OR “general practitioner” OR GP OR PCP OR “family medicine specialist”))) OR AB (((“primary care” OR “primary health care” OR medic* OR family OR community) W3 (physician OR doctor OR practitioner OR staff OR provider)) OR “general practitioner” OR GP OR PCP OR “family medicine specialist”)))	MA “palliative care” OR TI (((palliative OR terminal OR “end of life” OR hospice OR eol) W3 (management OR care OR medicine OR treatment))) OR AB (((palliative OR terminal OR “end of life” OR hospice OR eol) W3 (management OR care OR medicine OR treatment)))		MA “treatment barriers” OR TI (facilitat* OR enabler* OR motivat* OR assist* OR help* OR influenc* OR barrier OR bar* OR hinder OR hindrance* OR challeng* OR obstacle OR difficult* OR issue* problem OR hurdle OR imped* OR limit* OR deter* OR constrain* OR obstruct*) OR AB (facilitat* OR enabler* OR motivat* OR assist* OR help* OR influenc* OR barrier OR bar* OR hinder OR hindrance* OR challeng* OR obstacle OR difficult* OR issue* problem OR hurdle OR imped* OR limit* OR deter* OR constrain* OR obstruct*)

Filtered by: English language, Human July 1999 – December 2024

**Table:** Scopus

Population and context	Intervention	Context ⇓	Outcome
primary care physician	palliative care		facilitators and barriers
Title, Abstract and Keywords search ((“primary care” OR “primary health care” OR medic* OR family OR community) W/3 (physician OR doctor OR practitioner OR staff OR provider)) OR “general practitioner” OR gp OR pcp OR “family medicine specialist”	Title, Abstract and Keywords search ((palliative OR terminal OR “end of life” OR hospice OR eol) W/3 (management OR care OR medicine OR treatment))		Title, Abstract and Keywords search facilitat* OR enabler* OR motivat* OR assist* OR help* OR influenc* OR barrier OR bar* OR hinder OR hindrance* OR challeng* OR obstacle OR difficult* OR issue* AND problem OR hurdle OR imped* OR limit* OR deter* OR constrain* OR obstruct*

Filtered by: English, Human, 1999 July 01 – 2024 December 31

**Table:** Google Scholar

Population and context	Intervention	Context ⇓	Outcome
primary care physician	palliative care		facilitators and barriers
(((“primary care” OR “primary health care” OR medic* OR family OR community) AROUND3 (physician OR doctor OR practitioner OR staff OR provider)) OR “general practitioner”)	((palliative OR terminal OR “end of life” OR hospice OR eol) AROUND3 (management OR care OR medicine OR treatment))		(facilitat* OR enabler* OR motivat* OR assist* OR help* OR influenc* OR barrier OR bar* OR hinder OR hindrance* OR challeng* OR obstacle OR difficult* OR issue* AND problem OR hurdle OR imped* OR limit* OR deter* OR constrain* OR obstruct*)

Filtered by: English, 1999 – 2024 à Sorted by Relevance à Downloaded the first 100 references

**Table:** Policy Commons

Population and context	Intervention	Context ⇓	Outcome
primary care physician	palliative care		facilitators and barriers
("primary care physician" OR "general practitioner" OR "family doctor")	("palliative care" OR "end of life care" OR "terminal care")		(facilitator OR enabler OR assist OR help OR barrier OR challenge OR obstacle OR problem)

In Summary - Filtered by: English, 1999 – 2024 à Sorted by Relevance à Downloaded the first 100 references

**Table:** ProQuest Dissertations and Theses

Population and context	Intervention	Context ⇓	Outcome
primary care physician	palliative care		facilitators and barriers
("primary care physician" OR "general practitioner" OR "family doctor")	("palliative care" OR "end of life care" OR "terminal care")		(facilitator OR enabler OR assist OR help OR barrier OR challenge OR obstacle OR problem)

In Summary à Filtered by: English, 01.07.1999 31.12.2024 à Sorted by Relevance à Downloaded the first 100 references

## Appendix 2: key systematic reviews

**Table:** 

#	Author and year	
**1**	Abu-Odah et al., (2020)	Challenges on the provision of palliative care for patients with cancer in low- and middle-income countries: a systematic review of reviews.
**2**	Johnson et al., (2018)	General practice palliative care: patient and carer expectations, advance care plans and place of death—a systematic review
**3**	Mitchell, (2002)	How well do general practitioners deliver palliative care? A systematic review.
**4**	Mitchell et al., (2018)	Systematic review of general practice end-of-life symptom control.
**5**	Mitchell et al., (2020)	General practice nurses and physicians and end of life: a systematic review of models of care.
**6**	Peeler et al., (2024)	Primary palliative care in low- and middle-income countries: A systematic review and thematic synthesis of the evidence for models and outcomes.
**7**	Perdamaian et al., (Unpublished, currently underway)	Tentative title: The role of primary care practitioners in the provision of palliative care in the primary setting in low and middle income countries (LMICs): a mixed-method systematic review.
**8**	Rhee et al., (2020)	Facilitators and barriers to general practitioner and general practice nurse participation in end- of- life care: systematic review
**9**	Senior et al., (2024)	General practice physicians’ and nurses’ self- reported multidisciplinary end- of- life care: a systematic review.

## Appendix 3: data extraction form

**Table:** 

Author and year	Title	Aim(s)	Methods	Setting	Primary care physician description.	Other respondents in the study.	Aspect(s) of palliative care discussed

## Supplementary Information

Supplementary Material 1.

Supplementary Material 2.

## Data Availability

The corresponding author can provide data collection templates, data supporting the findings, and codes generated by this systematic review upon reasonable request.

## Abbreviations

BQ-II: Barriers Questionnaire II
LMIC: Low and Middle-Income Country
MMAT_18: Mixed methods appraisal criteria
PCP: Primary care physician
PICOS: Population, intervention, context, outcome, study design
PRISMA: Preferred reporting items for systematic reviews and meta-analysis
WHO: World Health Organization
